# Identification and genetic diversity analysis of hybrid offspring of azalea based on EST-SSR markers

**DOI:** 10.1038/s41598-022-18907-0

**Published:** 2022-09-08

**Authors:** Ming Su, Chunying Zhang, Shucheng Feng

**Affiliations:** 1 Shanghai Botanical Garden and Shanghai Engineering Research Center of Sustainable Plant Innovation, Scientific Research Center, Shanghai, 200231 China; 2 Shanghai Mjnicipal Landscape Management and Instructional Station, Shanghai, 200000 China

**Keywords:** Plant breeding, Plant genetics, Plant molecular biology, Plant reproduction

## Abstract

Azalea is a world famous flower with high economic and ornamental value. The breeding of new azalea varieties is mainly done by cross breeding. However, there is a risk that cross breeding may cause errors in the hybrid offspring due to contamination by exogenous pollen. Therefore, the identification of hybrid offspring is an important part of azalea breeding. In this study, the parents of three hybrid combinations and their 88 F1 hybrid offspring were selected to screen 15 pairs of EST-SSR primers to identify the authenticity of azalea hybrid offspring. The results showed that the authenticity of 88 azalea F1 hybrid progenies could be determined by at least four primer pairs. Genetic diversity analysis of azalea hybrid progeny revealed that the number of alleles and polymorphic information content of the progeny increased to different degrees, and the more distant the genetic distance between parents, the richer the polymorphic information. It is suggested that EST-SSR molecular marker can be applied for the early identification and genetic diversity analysis of the progeny of azalea hybrids. This method is of positive significance for improving the breeding efficiency of new varieties and exploring the genetic background of azalea.

## Introduction

Azaleas is an important group of the Rhododendron in the family Ericaceae, and famous flowers in the world^1^. It has important economic and ornamental values and occupies an important position in urban greening and landscaping^2^. At present, there are more than 1,000 species of Rhododendron and more than 30,000 varieties in the world^3–5^. Under natural conditions, the sexual reproduction of Rhododendron depends on visits from pollinators^6^. And Rhododendron varieties are bred mainly through various ways such as cross breeding, bud breeding and polyploid breeding, among which, cross breeding produces most of the new azalea varieties^7^. Azalea is a cross-pollinated woody plant with a long juvenile period, ranging from 3-10 years from sowing to flowering^8^, and it is susceptible to contamination by foreign pollen during thecross-breeding process, resulting in a mixture of true and false offspring. If these false hybrids cannot be screened and removed in time, it will certainly cause a lot of waste of land, manpower and material resources and reduce the reproductive efficiency. Therefore, the early identification of the authenticity of hybrids is a very important link in the breeding process of Azaleas.

With the continuous development of molecular technology, molecular markers have gradually appeared in the identification of hybrid progeny due to their rapid and accurate advantages. In particular, the third-generation molecular markers EST-SSR (Expressed Sequences Tags-Simple Sequence Repeats) is considered to be a relatively ideal method. Because of the advantages of codominant inheritance, good reproducibility and versatility, it is used for the identification of hybrids^9,10^. It is considered a more ideal method to identify hybrids^11^. At present, the EST-SSR molecular identification of hybrid progeny of woody plants such as *Litchi chinensis* Sonn.^12^ and *Camellia sinensis*^13^ has been well applied. However, current research on EST-SSR markers in Rhododendron is primarily focused on the development of molecular markers and genetic diversity analysis, and the use of EST-SSR molecular markers to identify hybrid progeny has not been reported^14–18^. On the one hand,There are just over two hundred est-ssr primers for different taxa with high polymorphism of Rhododendron, which is not enough to support more applications^14,16–18^. On the other hand, The polyacrylamide gel electrophoresis (PAGE) silver staining detection technique is mainly used to estimate the results by visual comparison of the size of DNA fragments with known molecular weight standards, and the accuracy and efficiency of this method limit the application of rhododendron EST-SSR molecular markers^19–21^. In this study, when faced with the identification of large numbers of hybrid offspring, the detection efficiency and precision of EST-SSR markers in Rhododendron were limited. Therefore, this study relies on EST-SSRmorphological markers and molecular markers to detect the geneticauthenticity of Rhododendron hybrid offspring, and usescapillary electrophoresis (CE) fluorescence detection technology to develop and select the EST-SSR core primers with potential for progeny identification.Exploring the potential of EST-SSR molecule we also explor the feasibility of EST-SSR marker technology in identifying the offspring of azalea hybrids, improving the efficiency of azalea hybrid breeding and providing technical support to accelerate the process of breeding new varieties of azaleas.

## Results

### Primer design and selection

A total of 2585 sequences were downloaded from the NCBI nucleic acid database for all azaleas ESTs and 436 redundant sequences were removed from the downloaded sequences using CD-HIT-ESTto obtain 2149 sequences non-redundant, MISA software was used to perform SSR loci screening^22^. A total of 233 eligible SSR-containing sequences were obtained and 258 SSR sites were identified, for 25 sequences contained more than one SSR locus, 13 of which were composite sequences. The total length of the sequence is 937.496 kb, averaging 3.63 kb base pairs, and has 1 EST-SSR site. The distribution of the different types of repeat is shown in Table 1. The analysis revealed that the highest number of repeat types are single nucleotides and dinucleotides, which represent 39.15% and 34.88%, respectively, followed by trinucleotide repeats, representing 22.09%, and some tetranucleotides (0.78%), pentanucleotide (2.33%) and hexanucleotide (0.78%). Among the different repetitive nucleic acid sequences, A/T (101) and AG/CT (85) are the repetitive motifs with the highest number of repeats, much higher than other repetitivemotifs.

**Table 1:** Distribution tables of different types of repetition.

RTs	Quantity	Percentage/ %	Repeat primitives (times)
Mononucleotide	101	39.15	A/T(101)
Dinucleotide	90	34.88	AC/GT(2);AG/CT(85);AT(3)
Trinucleotide	57	22.09	AAG/CTT(2);AAG/CTT(16);ACC/GGT(7);ACG/CGT(3); ACT/AGT(1);AGC/CTG(10);AGG/CCT(8);ATC/ATG(8); CCG/CGG(2)
Tetranucleotide	2	0.78	ACAG/CTGT(1);ACAT/ATGT(1)
Pentanucleotide	6	2.33	AAAAC/GTTTT(1);AAACT/AGTTT(1); AAGAG/CTCTT(2); ATCCC/ATGGG(1); CCCCG/CGGGG(1)
Hexanucleotides	2	0.78	AAAACC/GGTTTT(1); ACTGGC/AGTGCC(1)
Total	258	100.00	

Statistics of the length of the EST-SSR and the distribution of the repetition frequency of the Azalea motif found that the Mononucleotide motif and the Dinucleotide motif were more repetitive.The number of repeats for Trinucleotide motifs is less than 12, for Tetranucleotide motifs and Pentanucleotide motifs are less than 7, and for hexanucleotide motifs there are only 2 repeats of 4 and 5, see Table 2.

**Table 2:** Distribution of repeat of different primitives.

RTs	Number of primitive repetitions										
	4	5	6	7	8	9	10	11	12	$\ge 13$	Total
Mononucleotide	–	–	–	–	–	–	–	–	–	101	101
Dinucleotide	–	–	–	–	–	31	12	13	3	31	90
Trinucleotide	–	38	13	2	1	1	1	0	1	0	57
Tetranucleotide	1	0	0	1	0	0	0	0	0	0	2
Pentanucleotide	3	0	2	1	0	0	0	0	0	0	6
Hexanucleotides	1	1	0	0	0	0	0	0	0	0	2
Total	5	39	15	4	1	32	13	13	4	132	258

**Figure 1:**
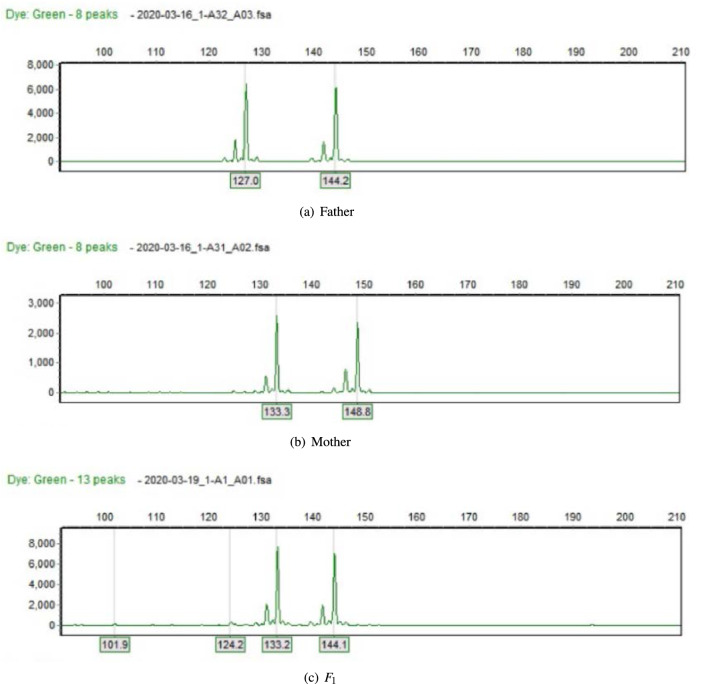
The heterozygous type.Peak value represents segment length.

**Figure 2:**
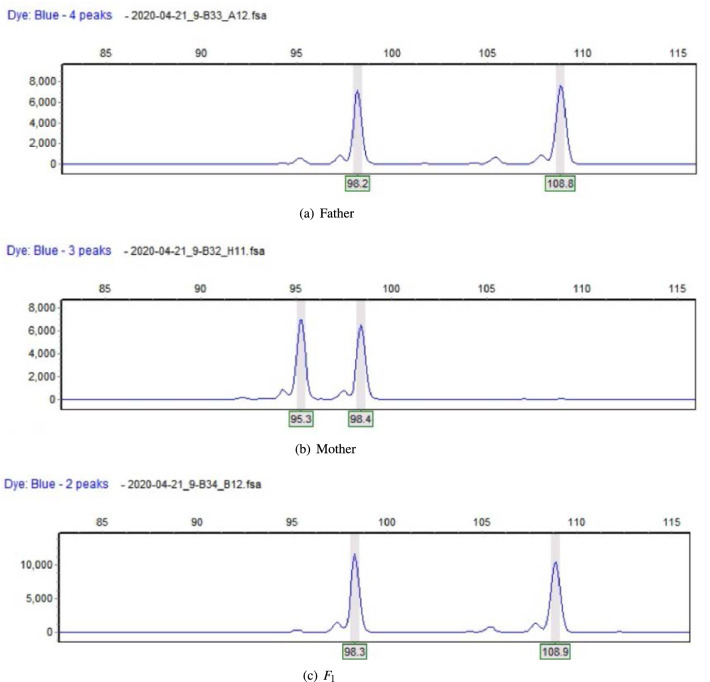
The paternal type.Peak value represents segment length.

Using the primer-blast for primer design, 18 SSR sites were finally selected. Among them, 2 single nucleotide repeats, 6 dinucleotide repeats, 3 trinucleotide repeats, 1 pentanucleotide repeat, 1 hexanucleotide repeat, and 5 complex repeats were designed. Three pairs of primers were designed for each SSR site, and a total of 54 pairs of primers were obtained. Then the invalid primers were removed by NCBI database comparison, and finally 19 pairs of primers were obtained.Together with 30 pairs of Azalea EST-SSR primers already published in the literature, a total of 49 pairs of primers were used to amplify DNA for all Azalea samples to be tested. Six types of bands appeared in the maternal paternal amplified bands: bbaa, bcaa, aaab, acab, ccab, and cdab. Analysis of the band pattern of 88 hybrid offspring revealed that the band pattern of the offspring could be divided into four types: heterozygous, paternal, maternal, and other types. Heterozygous type refers to offspring that contain specific bands from both parents, as shown in Fig. 1. The paternal type refers to the offspring that contain the father’s specific bands but not the mother’s specific bands as shown in Fig. 2. Maternal type means that the offspring contains parent specific bands, but not subspecies-specific bands; other types are relatively complex and some offspring have produced new bands.Among the above band types, only the parental type and heterozygous type could be used to identify the authenticity of the offspring, so 15 pairs of primers were selected from the 49 pairs of azaleas EST-SSR primers that had been screened as primers for authenticating the offspring of azaleas crosses., as shown in Table 3. Using these primers, the experimental results of 15 pairs of primers in 6 parents and 88 offspring can be obtained. 15 pairs of EST-SSR primers were used to amplify the DNA of 94 samples, and the result analysis is shown in Table 4. The percentage of polymorphics sites of 15 pairs of primers is 100%, total alleles is 95 , *Na* is 6.3333 and *Ne* is 3.9622. There is a large gap between the number of alleles obtained and the number of alleles observed and the number of effective alleles, indicating that the alleles are unevenly distributed in the population, *PIC* is from 0.8925 (S14) to 0.2400 (S9), the average value is 0.5767. Among them, the *PIC* for R13, R14, R15, R26, R28, S1, S10, S14 and S19 are $>0.5000$. The primers are highly polymorphic and can replace the core primers for progeny identification.

**Table 3:** Primer information EST-SSR 15 pairs of azalea.

Primer name	Sequence(5′–3′)		Motif	Tm/	Source
R13	Forward primer	ACGAGGAGAGGAGGCAAAAC	(AG)9	59.30	Meiqin Li, et al.^18^
	Reverse primer	TGATGGAGCCGACCTAAATG			
R14	Forward primer	TCTACTTTTCCCAACGCTCC	(CCA)10	58.50	
	Reverse primer	ACCCCCTTTCAATAGTCACC			
R15	Forward primer	ACTTTTCCCAACGCTCCTCT	(CCA)10	59.70	
	Reverse primer	CAAACCCTTAGCCAGTCCCA			
R26	Forward primer	CCCCAATCACTTGCCACTTT	(CT)17	58.70	Himanshu Sharma, et al^17^
	Reverse primer	TTTGGAGGAAGCGGCTAAGA			
R28	Forward primer	ATAGCCCCATGATCTAGTCTC	(CT)8	53.00	
	Reverse primer	GAGATTTTCTTGCCGTAGAAT			
R29	Forward primer	AGCAGACTATATGCAAAAGCA	(ATACAA)3	52.00	
	Reverse primer	TATCGCATGTTGGTTTAATTC			
S1	Forward primer	AGAAACAACCCTTGACCCTCC	(CT)17	59.81	Research group design
	Reverse primer	TTCTTACTCGTGTCTGGCGG			
S5	Forward primer	TACCACTTCCTGCTTCCCCT	(AGA)12	60.11	
	Reverse primer	AGGTCAACCGCCTTCATCTG			
S9	Forward primer	GAGGCCTTTATCCTCTCGGT	(ATAC)7(AG)20*	57.65	
	Reverse primer	ACTGTCAAGTCCAAGAAACC			
S10	Forward primer	TACTACTGCGCCAAAGCACT	(AG)31	59.49	
	Reverse primer	TTGGTCCCCTTCTGGTGATG			
S11	Forward primer	TACCACTTCCTGCTTCCCC	(AGA)12	58.70	
	Reverse primer	GTCAACCGCCTTCATCTGC			
S12	Forward primer	TGCTTCCCCTCCTCAATTCG	(AGA)12	59.79	
	Reverse primer	GATTGCCTGCGTCACTGAAC			
S14	Forward primer	ACACAATTCCACTTCAGGGCT	(CT)17	59.66	
	Reverse primer	TTGGAGGAAGCGGCTAAGAG			
S15	Forward primer	AATTCGGCACGAGGGAGAAG	(AAG)8	60.08	
	Reverse primer	CGCACCAACTAGCAAAACCC			
S19	Forward primer	GGTTTTCTAAGCCAGCCCCT	(CTT)9	60.11	
	Reverse primer	AATTGCAACCACTGCCAAGC

**Table 4:** Polymorphism of 15 EST-SSR primers.

Primer name	Na	Ne	Ho	He	H	I	PIC
R13	5	3.6894	0.5426	0.7328	0.7289	1.4309	0.6842
R14	3	2.8158	0.2766	0.6483	0.6449	1.0633	0.5688
R15	3	2.6586	0.5426	0.6272	0.6239	1.0320	0.5487
R26	10	8.6373	0.8830	0.8890	0.8842	2.2206	0.8728
R28	8	5.8633	0.8830	0.8339	0.8294	1.9214	0.8096
R29	2	1.9776	0.8723	0.4970	0.4943	0.6875	0.3722
S1	13	9.7906	1.0000	0.9027	0.8979	2.3534	0.8888
S5	7	1.8614	0.4255	0.4652	0.4628	0.9214	0.4255
S9	4	1.3726	0.3085	0.2729	0.2714	0.4854	0.2400
S10	6	2.7175	0.4149	0.6354	0.6320	1.3200	0.5987
S11	3	1.8854	0.4894	0.4721	0.4696	0.8055	0.4128
S12	3	1.7663	0.4362	0.4362	0.4339	0.7645	0.3884
S14	13	10.1041	1.0000	0.9058	0.9010	2.3802	0.8925
S15	12	1.8142	0.4362	0.4512	0.4488	1.1033	0.4353
S19	3	2.4796	0.7021	0.5999	0.5967	0.9763	0.5121
Average	6.3333	3.9622	0.6142	0.6246	0.6213	1.2977	0.5767

### Authentication of azalea hybrid offspring

The amplified bands of all the test samples were compared by fluorescence capillary electrophoresis. Based on the test results, it can be stated that all the test samples are true descendants. During the experiment, it was found that some primers performed well in some hybrid combinations, but did not work well in some combinations, indicating that the versatility of these primers was not sufficient, see Table 5. However, some primers have strong discrimination ability, such as primers S14, S1, R29, R28 and R26, which can distinguish more than half of the fertilized eggs in the three hybrid combinations and have good discrimination ability. Therefore, these 5 primer pairs are recommended as core primers for the identification of Azalea offspring. They can be used as center primers to complete the identification of the offspring based on the multiple marker combination method in detecting the authenticity of the hybrid offspring, as shown in Table 6. *R.pulcherum *‘Zihe’ *R.*‘Red Apple’ can use at least two primer pairs for offspring identification, and *R.* ‘Zibo’ *R. *‘Kirin’ and *R.pulcherum*‘Baihe’ *R.*‘Pink Bubble’ can be used for offspring identification. The offspring of these three crosses are true hybrids and can be identified by at least two pairs of primers.

**Table 5:** 15 Statistics on the number of EST-SSR azaleas.

Primer name	Identify the number of $$F_{1} $$			Total	Percentage/%
	*R.pulcherum*‘Zihe’ *R. * ‘Red Apple’	*R.* ‘Zibo’ *R. *‘Kirin’	*R.pulcherum*‘Baihe’ *R.*‘Pink Bubble’		
R13	0	0	18	18	20.45
R14	16	0	0	16	18.18
R15	17	0	7	24	27.27
R26	27	21	4	52	59.09
R28	15	21	27	63	71.59
R29	26	31	23	80	90.91
S1	29	28	27	84	95.45
S5	15	0	0	15	17.05
S9	0	0	0	0	0.00
S10	15	31	0	46	52.27
S11	15	0	0	15	17.05
S12	16	0	0	16	18.18
S14	29	31	26	86	97.73
S15	0	30	0	30	34.09
S19	0	25	0	25	28.41
Average	14.67	14.53	8.80	38.00	43.18

**Table 6:** Identification of Azalea subgeneration combined marker.

Hbridized combination	Primer pairs (primer pair combinations) that identify all $$F_{1} $$	Identify the minimum number ofprimers pairs used by all $$F_{1} $$
*R.pulcherum*‘Zihe’ R. ‘Red Apple’	(S14 & R14),(S14 & R15),(S14 & R26), (S14 & R29),(S14 & S1),(R26 & S1), (R26 & S10),(S1 & R14),(S1 & R14), (S1 & R15),(S1 & R15),(S1 & R28), (S1 & R29),(S1 & S10),(S1 & S11), (S1 & S12)	2
R. ‘Zibo’ R. ‘Kirin’	R29,S10,S14,(S15 & S1)	1
*R.pulcherum*‘Baihe’ *R.*‘Pink Bubble’	R28,S1,(S14 & R15),(S14 & R29)	1
All three combinations	(S1 & R26),(S1 & R28),(S1 & R29),(S1 & S10), (S14 & R15),(S1 & S15 & R14),(S1 & S15 & R15), (S1 & S15 & S11),(S1 & S15 & S12), (S1 & S19 & R14),(S1 & S19 & R15), (S1 & S19 & S11),(S1 & S19 & S12), (S14 & R14 & R28), S14 & R26 & R28), (R28 & R26 & S10),(R29 & R28 & R26), (R29 & R28 & S5), (R29 & R28 & S1), (R29 & R28 & S11),(R29 & R28 & S12)	2

### Genetic diversity analysis

Genetic diversity analysis was performed using 15 pairs of EST-SSR primers for the parents and offspring of the three hybrid combinations, respectively. The *R.pulcherum*‘Zihe’*R.*‘Red Apple’ combination amplified 56 bands, and each pair of EST-SSR primers produced an average of 3.7333 bands. By comparing the sizes of the amplified bands, it was found that 16 new bands different from those of the parents were generated. The combination of *R.*‘Zibo’*R.*‘Kirin’ amplified a total of 47 bands, and each primer pair amplified an average of 3.1333 bands. Table 7 shows a total of 11 new different parent bands. The combination of,*R.pulcherum*‘Baihe’ *R.*‘Pink Bubble’amplified a total of 51 bands, each primer pair had an average of 3.4 bands and a total of 14 different new bands from the parent.

**Figure 3:**
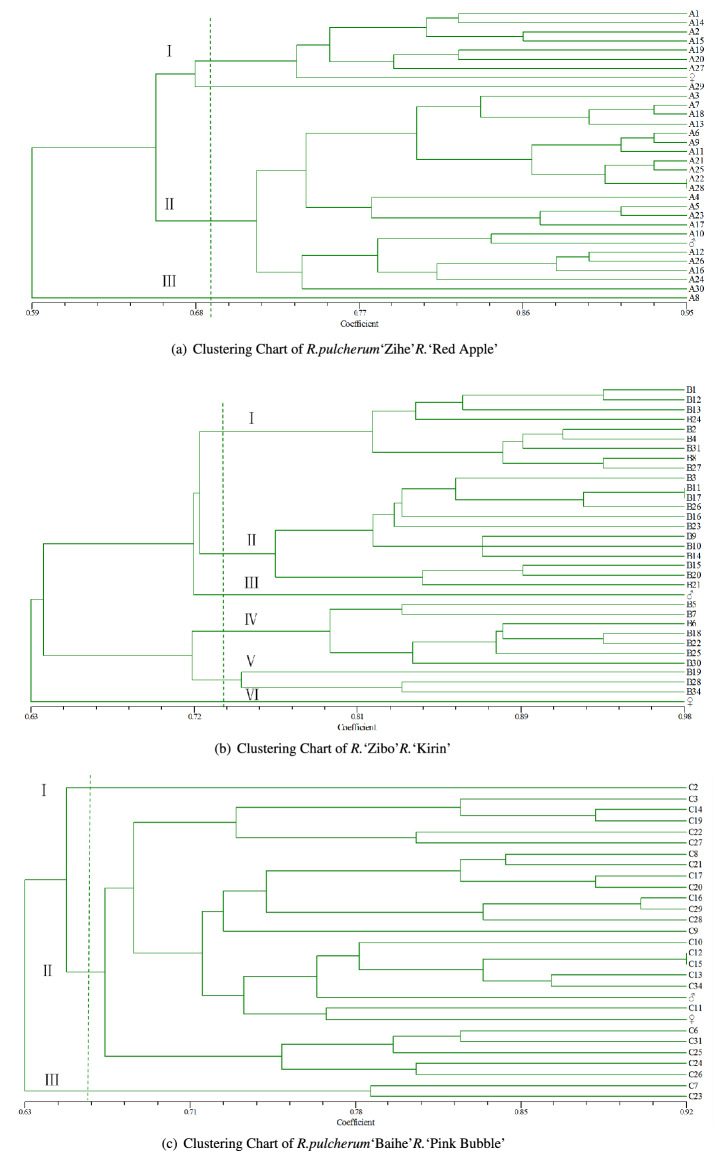
Cluster analysis diagram.

The cluster analysis of the three crosses shows that the combination of *R.pulcherum*‘Zihe’ *R.*‘Red Apple’ can be divided into three categories, with a similarity coefficient of 0.66, as shown in Fig. 3a. The first class had eight offspring, and clustered with their parents, representing 26.7% of the total. The first category had 8 offspring and clustered with the parent, which represents 26.7%, the second category had 21 offspring and clustered with the parent, accounting for 70%, and the third category was the offspring A8, a separate category. It can be seen that the offspring of this hybrid are more influenced by the parents, and the offspring appear to be paternally biased. It is noteworthy that both A8 and the parent are not clustered into one category, presumably more genetic mutations or recombination occurred during the cross. The *R.pulcherum*‘Baihe’ *R.*‘Pink Bubble’ combination can be divided into six groups at a similarity of 0.64, shown in Fig. 3c. Eight descendants form a group with the female parent, accounting for 29.6%, 6 descendants form a group with the male parent, accounting for 22.2% and the remaining 13.The genetic distance between the remaining 13 offspring and the parents is relatively large, accounting for 48.1%, indicating that a large number of genetic mutations or recombination’s occurred within this cross, resulting in more super-parental offspring. The combination *R.*‘Zibo’ *R.*‘Kirin’ can be divided into 3 categories, with a genetic coefficient of 0.68, shown in Fig. 3b. Twenty-one offspring were grouped with their parents, accounting for 67.7%, and the mother was groupedclustered with herself, and 10 offspring were groupedwith the parent, accounting for 32.3%. Another 10 offspring were clustered together, accounting for 32.3%. It can be found that the influence of the father on this cross is dominant.

**Table 7:** The bands of different combinations of primers.

Primer name	*R.pulcherum*‘Zihe’ *R.* ‘Red Apple’		*R.* ‘Zibo’ *R.* ‘Kirin’		*R.pulcherum*‘Baihe’ *R.*‘Pink Bubble’	
	Number of amplification bands	Number of additional bands	Number of amplification bands	Number of additional bands	Number of amplification bands	Number of additional bands
R13	3	1	2	0	3	0
R14	2	0	1	0	3	1
R15	2	0	2	0	3	0
R26	4	0	4	2	6	3
R28	4	1	5	1	4	0
R29	2	0	2	0	2	0
S1	7	3	6	2	4	0
S5	5	2	2	1	3	1
S9	4	2	1	0	2	0
S10	2	0	4	0	1	0
S11	3	0	2	0	2	0
S12	3	0	1	0	2	0
S14	5	1	4	0	5	1
S15	8	6	7	5	9	8
S19	2	0	3	0	2	0
Total	56	16	46	11	51	14

## Discussion

### Design and screening of 15 pairs of Azalea EST-SSR primers with potential for hybrid offspring identification

The experimental results revealed that EST-SSR primers with fragment repeat types of dinucleotide and trinucleotide are more advantageous in terms of versatility and polymorphism, which is consistent with the results of Mei-Qin Li^18^, so that the primers design process can preferentially select the type of dinucleotide repeat and EST-SSR trinucleotides. In the primer selection process, it was found that the 6 types of bands (bbaa, bcaa, aaab, acab, ccab, cdab) of the maternal paternal primers were recognized by potential of hybrid offspring, but the recognition efficiency of each type of band is different.. In this study, primers S1 and S14 were the cd ab band type,with the highest identification rate, 95.45% and 97.73%, respectively, indicating that the identification efficiency of the cd ab band type is the highest, and can be used as the first choice primer for the identification of hybrid progeny which is consistent with the results of Lei Yu et al^13^. In the process of offspring identification, using the least number of primers to identify more species in the direction of primer optimization^23^, which requires primers with good polymorphism and versatility. EST-SSR exists in the coding region of gene transcription, so the sequence is more conserved and universal. Although it is less polymorphic than Genomic-SSR primers, some studies have shown that there is no significant difference between the two in the evaluation of genetic diversity^24^. The *Na*, *I*, *PIC* of the 15 primer pairs investigated in this experiment were 6.3333, 1.2977, and 0.5767, respectively, showing aboundant polymorphisms. In particular, the five pairs of EST sequences of S14, S1, R29, R28, and R26 are generated Cdna clones randomly selected from different tissues and organs or at different developmental stages. If an EST-SSR is associated with a trait during the selection process, mark it may be related to the gene controlling this trait^25–27^ . For example, the EST-SSR involved in primer S1 comes from a cDNA library of relatively cold acclimated and non-cold acclimated Azaleas^28^. The relationship between this primer and the cold resistance of Azalea should be further explored. In addition, a Cdna library can be constructed for EST-SSR to screen other resistance traits of azalea, such as Rhododendron, such as salt resistance and high-temperature resistance. If the EST-SSR offspring identification is combined with marker-assisted selection technology to screen at the early stage of Azalea development, excellent offspring satisfying both authentic and target characteristics can be selected, thus greatly improving the breeding efficiency of Azalea.

### Only a minimum of four primer pairs are required to determine the authenticity of all crossed offspring of the three cross combinations in this experiment

In this experiment, EST-SSR fluorescent molecular markers were used to hybridize and identify all azalea samples tested. Because the traditional silver PAGE staining technique is tedious and time-consuming, especially in the face of large batches and multiple batches of experiments, it is prone to human error^21^, whereas the fluorescent capillary technique can accurately obtain the size of amplified fragment with high sensitivity and reproducibility, it is suitable for the detection of large numbers of hybrid offspring^29^ and can greatly improve the detection efficiency and accuracy^20^. The most conservative characteristics of EST-SSR make it more universal, which helps to reveal the genetic relationships between different genotypes^30,31^, and even has good versatility between different species, genera, and families^32–34^. This characteristic used to identify Azalea hybrid offspring are often effective in improving the efficiency of primer use. From the experimental results, it was found that the *R.pulcherum*‘Zihe’ *R.*‘Red Apple’ combination could be used for offspring identification with at least two primer pairs, *R.*‘Zibo’ *R.*‘Kirin’ and *R.pulcherum*‘Baihe’ *R.*‘Pink Bubble’ both cross combinations can be identified with 1 pair of primers for the offspring. In contrast, only 2 pairs of primers were required for the complete identification of all the hybrid offspring in these 3 cross combinations, indicating that the screened primers have good generality among the 3 cross combinations. In order to ensure the reliability of the results, each hybrid offspring must be identified at least twice to determine the true hybrid^35,36^, so this study requires four pairs of primers were to complete the accurate identification of all hybrid offspring in this study. The use of EST-SSR fluorescent molecular markers has also been proved to be a feasible for the identification of Azalea hybrids and has important value inr improving breeding efficiency. However, due to the limited number of samples involved in this experiment, the identification ability to identify primers can only be limited. In the future, we can continue to expand the test on this basis by adding more hybrid combinations and hybrid offspring to verify the generality and discriminatory ability of the primers.

### The more distant the parents are, the richer the diversity of the offspring

This study found that compared to the genetic diversity of the parents, the number of alleles and polymorphic information content of the offspring showed different degrees of increase. When comparing the amplified band sizes of the hybrid offspring, it was found that the offspring of all three hybrid combinations showed new bands and deletion of both parents’ loci, except for the bands containing the parents, especially the more distantly related *R.pulcherum*‘Zihe’ *R.*‘Red Apple’ combination, which produced The number of new bands was the highest in the *R.pulcherum*‘Zihe’ *R.*‘Red Apple’ combination. This situation is also widespread in woody plants such as *Litchi chinensi*s Sonn.^13^ and *Camellia sinensis*^37^, probably because the polymorphism of simple repeat sequences is based on differences in the number of repeats in the amplified region, due to mutations within the primer binding region produced variation in the null allele, while mutations between primer regions may lead to the generation of new alleles. Therefore, it is presumed that these three hybrid combinations formed new genotypes during the hybridization process because of genetic recombination. Therefore, bands of different lengths were produced when the DNA was amplified with the same primers^13^. From the cluster analysis, it is found that the parental genetic distances of three cross combinations are different. The combination of *R.pulcherum*‘Zihe’ *R.*‘Red Apple’ and *R.*‘Zibo’ *R.*‘Kirin’ is more affected by parental genetic influence, with more partial paternal genetic offspring, and parental genetic distance close to *R.pulcherum*‘Baihe’ *R.*‘Pink Bubble’ combination appeared more superparent offspring. This may be due to large genetic differences between parents inhibiting the chromosomal recombination of the offspring when the genetic differences between the parents are too large, resulting in biased segregation. All three cross combinations showed more extensive genetic variation in the offspring with rich genetic diversity, and the rich genotypic variation may cause phenotypic diversity^37^. In particular, the offspring of the most distant relatives, such as A8, C7, and C23, were selected as favorable breeding intermediate materials for the next step of genetic improvement.

## Materials and methods

### Plant material

The chromosome number of the *Rhododendron* subg. *Tsutsusi* was 2n = 26 with x = 13 as the base chromosome number, which is the one of the frequently encountered base chromosome numbers in Azalea^38^. The test samples were the parents of three sets of hybrid combinations of azalea varieties *R.pulcherum*‘Zihe’ *R.*‘Red Apple’,*R.pulcherum*‘Baihe’ *R.*‘Pink Bubble’ and *R.*‘Zibo’ *R.*,‘Kirin’ and the 88 stably flowering F1 hybrid offspring that differed somewhat from the parental morphology,see Table 8. They were collected in April 2019 from the Wanjing Rhododendron Breeding Garden in Beilun, Ningbo, Zhejiang Province, China. Three to four fresh and intact young leaves from the current year branches were collected and returned to the laboratory, dried and stored in silica gel desiccant, and used as material for DNA extraction.

A modified CTAB method was used to extract DNA from Azalea leaves, NanoDrop2000 and Pico Green were used to detect DNA purity and concentration, and 1% agrose gel electrophoresis was used to detect DNA integrity.

**Table 8:** The situation of 3 hybrid combinations.

Hbridized combination	Parents	Varieties name	Origin	The number of F1
1	Father	*R.* ‘Zihe’	China	30
	Mother	*R.*’Red Apple’	America	
2	Father	*R.pulcherum.*‘Zibo’	China	31
	Mother	*R.*‘Kirin’	Japan	
3	Father	*R.pulcherum* ‘Baihe’	China	27
	Mother	*R.* ‘Pink Bubble’	America	

### EST-SSR marker

All Rhododendron-related EST sequences were downloaded from the NCBI nucleic acid database (as of November 2019), use CD-HIT-EST online software$(http://weizhong-lab.ucsd.edu/cdhit_suite/cgi-bin/index.cgi?cmd=cd-hit-est)$ to delete redundant sequences from the downloaded sequences, use MISA online software$(https://webblast.ipk-gatersleben.de/misa/)$MIAS to screen SSR sites, use primer blaster for primer design, use oligo7 screening primers to ensure that there are no hairpin structures, neither dimers nor mismatches^22^. The designed primers were checked by the blast match in the NCBI database to remove invalid primers. In addition to the self-designed primers, 30 EST-SSR Azalea primers were selected from the published literature for evaluation. According to the experimental results of the relationship analysis and progeny identification of azalea, universal primers with clear bands and good polymorphism were screened for primer synthesis and capillary flouresence electrophoresis. Capillary electrophoresis was performed using an ABI3730xl sequencer, and the results were read and analyzed using Gene Marker V 3.0.1^39^. The results of the fluorescence capillary electrophoresis experiments were read using Gene Marker V 3.0.1. By comparing the difference between the flouresence signal of the amplified fragments and the molecular weight standard, the fragment size in a single signal is obtained proportionally and saved in Excel. Data Formater is used to convert the raw data into a data format readable by the analysis software^40^, and Popgen32 and Power Marker software were used to analyze Number of alleles( *Na*), Number of effective alleles (*Ne*), Observed heterozygosity (*Ho*), Expected heterozygosity (*He*), Nei’s genetic diversity index (*H*), Shannon’s information index (*I*) and Polymorphic information content (*PIC*)^41^.

### Additional information

All plant materials are cultivated species, not wild or endangered plants. Experiment comply with the IUCN Policy Statement on Research Involving Species at Risk of Extinction and the Convention on the Trade in Endangered Species of Wild Fauna and Flora. Experimental research ,including the collection of plant materials, comply with relevant institutional, national, and international guidelines and legislation. All plant materials belong to Shanghai Botanical Garden and are kept in Wanjing Rhododendron Breeding Garden, and the use of these materials was approved by the above two institutions.

## Data Availability

The datasets generated during and analysed during the current study are available from the corresponding author on reasonable request.
